# *SPACIA1/SAAL1*: a newly identified gene associated with aberrant proliferation of synovial fibroblasts

**DOI:** 10.1186/ar3662

**Published:** 2012-02-09

**Authors:** Tomoo Sato, Ryoji Fujii, Koji Konomi, Naoko Yagishita, Satoko Aratani, Natsumi Araya, Hiroyuki Aono, Kazuo Yudoh, Noboru Suzuki, Moroe Beppu, Yoshihisa Yamano, Kusuki Nishioka, Toshihiro Nakajima

**Affiliations:** 1 St. Marianna University School of Medicine, Kawasaki, Japan; 2 Santen Pharmaceutical Co., Ltd., Osaka, Japan; 3 Institute of Medical Science, Tokyo Medical University, Tokyo, Japan; 4 Misato Marine Hospital, Kochi, Japan

## Background

The bone and cartilage destruction seen inrheumatoid arthritis (RA) is caused by synovial pannus formation, which is characterized by aberrant proliferation of synovial fibroblasts. Inhibition of synovial proliferation has recently been reported to be a promising therapeutic strategy for RA.However, the specific mechanism underlyingdysregulated proliferation of synovial fibroblasts remains unclear.

## Objective

We aimed toidentify and characterize genesthat are involved in the aberrant proliferation of synovial fibroblasts.

## Methods

Microarray analysiswas performed to identifythe genes that had upregulated expression inmice with collagen-induced arthritis (CIA). The effect of candidate genes on the proliferation of synovial fibroblasts was screened using antisense oligodeoxynucleotides and small interfering RNAs (siRNAs).

## Results

We identified a novel gene named *SPACIA1/SAAL1 *(synoviocyte proliferation-associated in CIA 1/serum amyloid A-like 1)that was associated with aberrant proliferation of synovial fibroblasts. Immunohistochemicalanalysis indicated that SPACIA1/SAAL1 was strongly expressed in the foot joints of mice with CIA and in the thickened synovial lining of the human RA synovium. Transfection of siRNA targeting *SPACIA1/SAAL1*into RA synovial fibroblastscould inhibit tumor necrosis factor (TNF)α-induced proliferation more effectively thanit could inhibit serum-induced proliferation.In addition,the antiproliferative effect of *SPACIA1/SAAL1 *siRNA was caused byinhibition of cell cycle progression and not by induction of apoptosis.We established transgenic (Tg) mice that overexpressed SPACIA1/SAAL1. These Tg mice did not spontaneously develop arthritis or cancer. However,inducing CIA causedgreatersynovial proliferation and worse diseasein Tg mice thanin wild-type mice.

## Conclusion

SPACIA1/SAAL1 plays an important role in the aberrant proliferation of synovial fibroblasts under inflammatory conditions.

